# Osgood-Schlatter Disease: Appearance, Diagnosis and Treatment: A Narrative Review

**DOI:** 10.3390/healthcare10061011

**Published:** 2022-05-30

**Authors:** Francisco Corbi, Sergi Matas, Jesús Álvarez-Herms, Sebastian Sitko, Ernest Baiget, Joaquim Reverter-Masia, Isaac López-Laval

**Affiliations:** 1 Institut Nacional d’Educacio Fisica de Catalunya (INEFC), Universitat de Lleida (UdL), 25192 Lleida, Spain; f@corbi.neoma.org (F.C.); smatasg@gencat.cat (S.M.); 2 Physiology and Molecular Laboratory (Phymo Lab), 40170 Segovia, Spain; jesusah80@gmail.com; 3 Faculty of Health and Sport Sciences (FCSD), University of Zaragoza, 22002 Huesca, Spain; sebastiansitko@yahoo.es; 4 National Institute of Physical Education of Catalonia (INEFC), University of Barcelona (UB), 08007 Barcelona, Spain; ernest.baiget@uvic.cat; 5 Departament de Didactiques Especifiques, Universitat de Lleida (UdL), 25003 Lleida, Spain; joaquim.reverter@udl.cat

**Keywords:** Osgood-Schlatter, knee pathology, patellar tendon, patellar tendonitis, tibial tuberosity, apophysitis

## Abstract

Osgood-Schlatter disease is the most common osteochondritis of the lower limb in sport-practicing children and adolescents. Its manifestation usually coincides with the appearance of the secondary ossification center of the tibia and is linked to the practice of sports with an explosive component. In the present study, a review of the factors related to its appearance, diagnosis and treatment was carried out. Its appearance seems to be multifactorial and related to multiple morphological, functional, mechanical and environmental factors. Given all the above, risk factor reduction and prevention seem the most logical strategies to effectively prevent the appearance of the condition. In addition, it is essential to create prevention programs that can be objectively assessed and would allow to stop the progress of the pathology, particularly in those sports where high forces are generated on the insertion zone of the patellar tendon at sensitive ages. More studies are needed to clarify which type of treatment is the most appropriate—specific exercises or the usual care treatment.

## 1. Introduction

**Figure 1:**
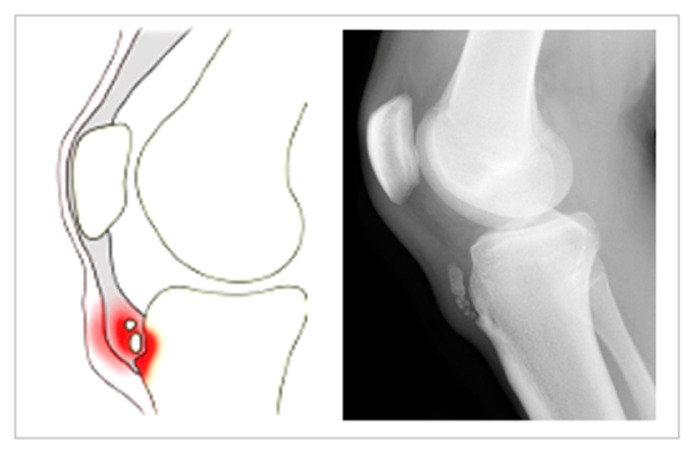
Illustration of the typical features of Osgood-Schlatter disease.

Osgood-Schlatter disease (OSD), also known as Lannelongue disease [1], is a type of osteochondrosis first described by Osgood and Schlatter in 1903 [2]. It consists of the onset of a traction apophysitis as a consequence of repeated contractions of the femoral rectum part of the quadriceps [3] (see Figure 1) and may be bilateral [4]. OSD is one of the most common overuse of the lower limb injuries among children and adolescents [5] and is usually a self-limiting pathology [6]. Its manifestation coincides with the development of the secondary ossification center of the anterior tibial tuberosity (apophyseal phase), which usually occurs at around age 9 in girls and 11 in boys. However, symptoms usually first appear between ages 8 and 12 among the former, and between 12 and 15 among the latter [7]. Despite this, cases in adults have been reported [8]. OSD has been associated with other pathologies and alterations, such as compartment syndrome, meniscal and patellar tendon injuries [9] or hyperactivity and attention deficit [10]. In addition, nearly 40% of patients reported pain after long-term follow up, which may lead to consequences such as the chronification of knee pain and the appearance of tendinosis, which, in turn, will end up conditioning the application of surgical techniques [11].

Given the social and economic repercussions of this pathology, the main objective of this review is to synthesize the factors related to its presentation, diagnosis and treatment.

## 2. Material and Methods

### 2.1. Information Sources

A computer-based scientific literature search was completed from inception to 28 February 2022, using the following information sources: Medline (PubMed), Web of Science (WOS), the Cochrane Collaboration Database, Cochrane Library, Evidence Database (PEDro), Evidence Based Medicine (EBM) Search review, National Guidelines, EMBASE, Scopus and Google Scholar system. It used the keywords: “Osgood-Schlatter”, “epidemiology”, “etiopathology”, “symptomatology”, “diagnosis”, “treatment” and “sport”, with Boolean operators such as: “AND” or “OR”. The specific search strategy was performed following the Boolean equation: (“epidemiology” [All Fields] OR “epidemiology” [All Fields]) AND (“etiopathology” [All Fields] OR “etiopathology” [All Fields]) AND (“symptomatology” [All Fields] OR “symptomatology” [All Fields]) AND (“diagnosis” [All Fields] OR “diagnosis” [All Fields]) AND (treatment [All Fields] OR treatment [All Fields]) AND (“Osgood-Schlatter” [MeSH Terms] AND “sports” [All Fields]). Through this equation, the relevant articles in this field were obtained, applying the snowball strategy. Furthermore, this narrative review was conducted in accordance with the preferred reporting items for review statement guidelines [12].

### 2.2. Study Inclusion Criteria

All titles and abstracts from the search were cross-referenced to identify duplicates and any potential missing studies. The titles and abstracts were screened for a subsequent full-text review. The search for published studies was independently performed by two authors and disagreements about all outcomes were resolved through discussion. The criteria for allocations in the articles were satisfied. A manuscript’s full-text was obtained to ascertain if the publication satisfied the inclusion criteria. In addition, the reference sections of the selected articles were searched to identify other relevant articles. Finally, for the current review, only studies focusing on OSD in relation to the multifactorial and multiphenotypic disease framework in sport practicing were included.

### 2.3. Study Exclusion Criteria

Duplicated articles were deleted. Furthermore, abstracts, non-peer-reviewed papers and book chapters were excluded. To effectively quantify the effectiveness of the scientific evidence, the search carried out was converted to categories of epidemiology, etiopathology, symptomatology, diagnosis and treatment. The practical applications were also referenced at the end of the main sub-categories.

## 3. Results

**Figure 2:**
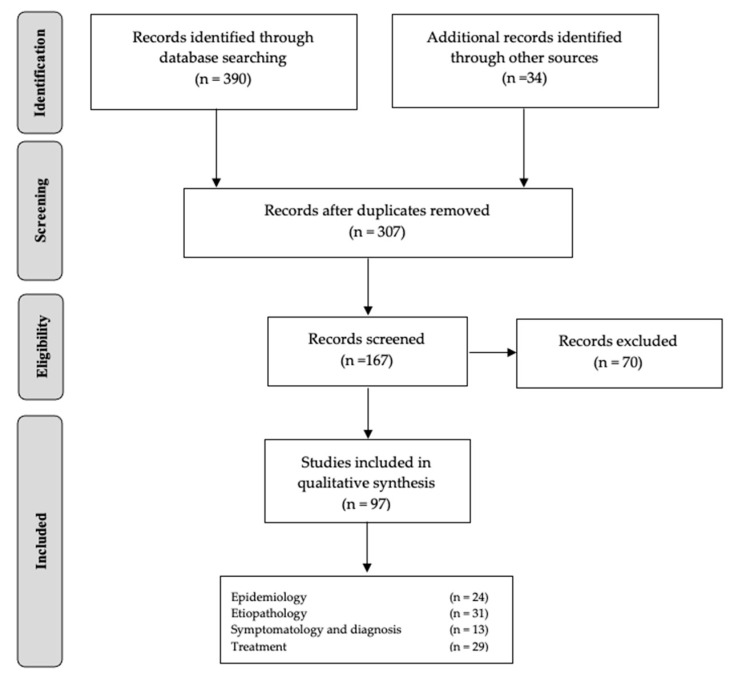
Flow diagram of the study selection.

The initial search of the literature detected 424 articles about ODS disease; nevertheless, 212 were excluded after being determined unrelated to the inclusion criteria or to the main keywords determined for this narrative review, or both (Figure 2). In relation to the main sub-categories, the structure of the document was established as follows: epidemiology, 24 articles; etiopathology, 31 articles; symptomatology and diagnosis, 13 articles; and treatment, 29 articles.

## 4. Epidemiology

The prevalence of OSD ranges from 6.8% to 33% [13,14,15], affecting 1 in 10 athletic adolescents [3], and depends on factors such as the degree of development, the sport discipline or the presence of preventive programs [6]. For example, Lucena et al. found a prevalence of 9.8% among males and females (mean age 13.7 years) [8], while Kujala et al. reported a prevalence of 12.9% (mean age: 13.1 years) [9]. Furthermore, it seems that symptoms increase as bone maturation progresses and that the onset does not depend on the hours of sports practice [16]. Symptoms can persist until adulthood in 10% of cases [17]. Additionally, between 20% and 30% of all cases are bilateral [18].

### 4.1. Gender

The ratio of cases between males and females is 14:1 [19]. Furthermore, Kaneuchi et al. found different prevalence peak ages according to gender. When no gender distinction was made, the peak age was found to be 12 (prevalence of 13.8% for boys and 11.4% for girls). However, among girls, the peak was between 9 and 10 (9.2–10.9%), while among boys it was 14 (10.3%). This fact could be explained due to the fact that girls reach the bone maturation stage of the tibial tuberosity two years earlier than boys [16]. Furthermore, some authors suggest that the risk of developing OSD is higher among females during the epiphyseal stage, particularly among sports practitioners [15].

### 4.2. Practice Level

Lucena et al. [8] calculated a prevalence of 13% among adolescents who practiced sports and 6.7% among those who did not. In comparable groups, Kujala et al. [9] found a prevalence of 21% and 4.5%, respectively. The previous findings seem to indicate that the incidence is higher among the sport-practicing adolescent population.

### 4.3. Sports

OSD is the knee pathology with the highest incidence among adolescent soccer players [20] and accounts for 13.6% of all knee pathologies in soccer players aged 12 to 15 [21]. Furthermore, it is bilateral in 20–30% of cases [22,23]. It has also been reported among practitioners of other sports that feature explosive, anaerobic and acyclic components and continuous changes of direction, such as basketball, sports gymnastics, volleyball, karate, taekwondo, baseball and running, as well as among people who practice multiple sports [24,25,26,27].

### 4.4. Bilaterality of the Disease

Although the etiology is not clear and the causes are still unknown, a common hypothesis is that the asynchronous development of bone and soft tissue during the maturation stage (especially of the femoral rectum part of the quadriceps) generates imbalances [28]. Alterations in traction forces are considered a trigger for OSD because the force levels that increase considerably in certain growth phases can generate imbalances. This increases stress in the joint and generates bilateral asymmetry between limbs, which exacerbates the incidence of this pathology [29].

## 5. Etiopathology

The appearance of OSD seems to be multifactorial and encompasses mechanical, functional, morphological, environmental and psychosocial factors [30,31].

### 5.1. Mechanical Factors

Most authors believe that the main cause is sustained traction of the patellar tendon and the repetitive strain caused by a strong and violent pull of the muscle and the patellar tendon applied on the apophyseal cartilage of the anterior tibial tuberosity [32], along with change that happen during a growth spurt [33]. The stress loads transmitted from the quadriceps to the cartilage through the quadriceps and patellar tendons end up causing a cartilaginous avulsion, which, as the ossification center tends to consolidate, will evolve towards the creation of bone tissue. Thus, for example, a higher incidence of OSD has been found in the support leg due to the tractional loads applied when the quadriceps contracts eccentrically [26]. Occasionally, bone fragmentation may be observed in the late evolutionary stages [17]. In patients affected by OSD, Enomoto et al. observed a lower deformation capacity of the patellar tendon per unit of tractional force [34]. This highlights the mechanical and anatomical causes associated with specific situations (patella alta, patella infera, shortening of the rectus femoris or quadriceps muscle tightness/inflammation in the tendon) as key elements in the appearance of OSD due to segment avulsion [35,36].

Despite all this evidence, some authors also determined that OSD is a tendinous injury and not an avulsion fracture. It appears, according to this other causal factor theory, that it begins in the apophyseal stage and looks like an apophysiolysis compared to an epiphysiolysis, only representing a precondition before the impact of pathologic stress forces without signs of osteonecrosis [37].

### 5.2. Functional Factors

Firstly, the strength level decompensation between the quadriceps and the hamstrings should be highlighted, given the existing agonist–antagonist relation between both muscles [38,39]. Several other highly influential factors are the type of movement pattern (jumping actions, sprints, kicks and changes of direction), increase in muscle mass and strength levels which appear in the pubertal period (especially among boys), flexibility reduction in the quadriceps muscle [8] and application of intensive (high training loads in a short time) and highly specific (repetition of movement patterns in which high inertial loads are generated) training. Furthermore, it appears that increased tension in the rectus and biceps femoris, gastrocnemius and soleus may also play a role in the etiopathology [1]. Regarding the hamstrings, it seems that the shortening of this muscle group could affect the lever arm, the torque and the compression of the patella between 30 and 60° of knee flexion. This might explain why quadriceps functionality has been associated with OSD [40,41]. Recently, Itoh et al. [42] compared fourteen types of movement patterns and found that the patterns that generated the greatest moments of force in OSD patients were the one leg drop (jump with a single-leg landing), braking actions (start-stop) and changes in direction (cutting). Moreover, the maximum angular impulse was reached during changes in direction, one-leg drops and in braking actions. Additionally, in sports such as soccer, it seems that adopting a delayed center of gravity (CG) position when kicking the ball could increase the risk of OSD. This is due to the pelvic retroversion that occurs in this situation and the increased tension that must be supported by the quadriceps due to its biarticular muscle condition [9]. Enomoto et al. [43] suggest that a stiffer rectus femoris under stretched conditions (45° and 90° flexion) is linked to OSD.

### 5.3. Morphological Factors

The main risk factors for the support leg appear to be height, weight, body mass index (BMI), loss of flexibility in both legs, height of the internal longitudinal arch of the support foot (risk increases with taller arch), previous diagnosis of Sever’s pathology [44], reduction of the ankle dorsal flexion of 10° or less [40], tibial rotations (an increase in the condyle-malleolar angle and tibial external rotation) [45,46], concomitant genu valgum and pronated foot [47] and lateral patellofemoral maltracking [48]. In patients analyzed with Nuclear Magnetic Resonance (NMR) techniques, Demirag et al. [30] found that when the patellar tendon was inserted more proximally and in a larger area of the tibia, the risk of OSD increased. Furthermore, Green et al. [49] recently found a direct relationship between a greater posterior tibial slope angle and a higher incidence of OSD and Pan et al. [50] demonstrated a higher Insall–Salvati Index in non-operative OSD patients.

Regarding the patella, though no clear consensus exists [51], it seems that morphology, stability, position and the fact that it is bipartite [23] could have an important influence [40,52,53], since all these would modify the moment of force generated on the patellar tendon insertion. While performing several autopsies, Ehrenborg also observed that the stress generated by the patellar ligament fibers could influence OSD, especially when they were shortened [54]. Conversely, no significant relationships were observed with patellar congruence and inclination angles, Q angle or patellar height [45,55]. Recently, Sheppard et al. [56] demonstrated that patients with OSD usually have an increased posterior tibial slope angle.

### 5.4. Environmental Factors

The appearance of OSD may be induced by factors related to the management of training loads, such as the intensity level during physical exercise, volume [54] and its modification [57,58], early specialization [56] and even with certain dietary deficiencies, such as vitamin D deficiency, particularly in countries with few hours of sun [59,60]. It also seems that the incidence is greater during the winter, especially in cold countries. Vitamin D plays an essential role in calcium phosphate homeostasis, and its deficiency leads to disruption in the growth plate organization. For these reasons, some authors have theorized about the possibility that fragility in the tibial tuberosity could appear in adolescents with vitamin D deficiency, which would compromise the enthesis’ mechanical response. Although this theory has yet to be confirmed, when children with OSD and vitamin D deficiency took a supplement, symptoms disappeared [60].

### 5.5. Psychological Factors

Reassurance and family education seem to be key components of successful treatment, management and recovery of OSD. The occasionally long duration of OSD symptomatology—sometimes over a year—could have repercussions on the way the patients perceive the injury and recovery. In most sports, it is known that long-term injuries, in which the patient experiences pain, produce worsened sleep, increased anxiety, heightened catastrophizing and low mood, especially among young athletes [61,62]. In this regard, the support of family and the health professionals who treat the athlete is essential. In a study conducted among health professionals treating OSD, 85% of those surveyed considered that psychological factors were important for returning to activity [31].

## 6. Symptomatology and Diagnosis

The main symptom of OSD is pain of variable intensity which increases when the site is pressed, especially in positions such as kneeling [63]. Furthermore, inflammation and hypersensitivity are commonly found in the anterior tibial tuberosity, where the patellar tendon inserts. This can be observed especially during physical and sports practice and might manifest as a limp. During the acute phase, symptoms usually evolve gradually from light and occasional to severe and continuous pain [8,17]. Thickening of the patellar tendon insertion can be noticed upon palpation, and is often accompanied by pain, particularly when resisted knee extensions or counter-resisted flexions are performed [24]. The pain is usually linked to an increase in blood flow to the area, which over time may cause neo-vascularization [64]. Guldhammer et al. [65] found that the median duration of OSD was 90 months (interquartile range, 24–150 months), with 42.9% of patients reporting daily pain. According to Kaya et al. [66], approximately 50% of patients fully recovered 2 years after being diagnosed, though levels of extensive strength continued to be lower. The differences between the studies could be explained by factors such as the type of sport practiced, patient age at the time of the event, methodology used and various environmental and cultural factors. Despite this, some cases may become chronic and complications such as pseudoarthrosis, genu recurvatum, patella alta, fragmentation–migration of bone fragments and a reduced knee flexion [4] or even osteochondromatosis may appear [67].

OSD diagnosis is mostly clinical and based on symptoms [33]. However, it should be confirmed by complementary radiological tests (X-ray, ultrasound or MRI), which allow OSD to be differentiated from other types of pathologies, such as fractures, tumors and infections, tendinitis or Hoffa’s disease [16,24,68].

### 6.1. Conventional Radiology

Usually, this is the first-choice complementary technique to rule out other pathologies, especially if its presentation is severe or atypical [69]. A sagittal plane of the knee with thigh rotation of 10–20° allows for easy identification of irregularities and separations of the apophysis from the tibial tuberosity, especially in early stages. In more advanced stages, it allows to identify bone fragmentation [40]. In general, X-rays allow for the establishment of three different levels of involvement: Grade (I), slight tuberosity elevation; Grade (II), radio lucidity of the tuberosity; and Grade (III), tuberosity fragmentation. On the other hand, some cases are asymptomatic, despite structural alterations being observed during radiological examination [70].

### 6.2. Ultrasound

Ultrasound is used in OSD diagnosis and monitorization given it is non-invasive, reliable, fast and low cost. It allows to visualize the fragmentation of the ossification center, patellar tendon injuries, the presence of edema and the appearance of possible reactive bursitis [16,39,71].

### 6.3. Nuclear Magnetic Resonance (NMR)

Possibly the most sensitive technique for diagnosis, since it enables the visualization of cartilage and the detection of edema, even before an ossification center tear occurs [72]. Furthermore, it is key for the early detection of the pathology [73]. Unfortunately, its high cost usually limits its use to cases in which the previous techniques prove insufficient. Hirano et al. [74] described five stages of OSD based on this technique: Stage 0—NMR is normal, although the patient may present certain symptoms; Stage 1 or early stage—no signs of inflammation visible in radiological exploration; Stage 2 or progressive stage—the torn secondary ossification center can be observed; Stage 3 or terminal stage—the ossicle has been completely separated and thickening of the tendon appears; and Stage 4 or healing proliferation of new bone tissue is observed.

Recently, other objective methodologies, such as algometry or thermography, have been used as diagnostic tools, with promising results [75].

## 7. Treatment

Given that symptoms usually subside over time, that the secondary ossification center tends to ossify, and that approximately 80% of patients usually recover. The disorder generally resolves with skeletal maturity and the treatment is normally conservative [24,33]. OSD usually resolves spontaneously when the affected area completes its growth, around Risser Stage 1 [57]. Symptom duration remains unclear: Rathleff et al. [76] recently reported high rates of successful outcomes among OSD patients (80% at 12 weeks and 90% after 12 months), with 16% having returned to sport after 12 weeks, and 67% after 6 months. However, Krause et al. [32] reported that 90% of OSD patients treated with conservative treatment had fully recovered from symptoms in approximately one year, although the strength level and functionality deficits may be maintained over time.

Several treatment strategies have been proposed: a decrease in physical activity [1,77], application of cold, the use of knee orthoses that pressure the patellar tendon to reduce tractional load on the insertion [78], physical therapy [6,79], warm-up and cool-down exercises before and after training and competition [80] and stretching of the leg extensor musculature to reduce the tension generated by the extensor apparatus [6,24,57,81]. For the latter, it is essential to avoid excessive tension on the insertion. Furthermore, Ross et al. [58] recommend that flexibility be improved in the hamstrings, gastrocnemius and the iliotibial band. In extreme cases, cast immobilization has been considered [1], although this technique might generate atrophy of the quadriceps musculature and structural disorganization.

Regarding activity type, it is recommended that any running, jumping and changing of directions should be reduced or eliminated until symptoms improve. These activities can be replaced by swimming and pedaling, which do not significantly increase tendon load [82]. Furthermore, some authors recommend core stabilization exercises, given that reduced core stability is linked to increased peak torque in knee flexion during the stance phase of running [83]. Additionally, increased core stability has been associated with better knee functionality in jumping actions [84], and education on activity modification and progressive knee-strengthening exercises appear to be effective [32]. Despite these potential benefits, more studies are needed to clarify which type of treatment is the most appropriate. According to Neuhaus et al. (2021), no specific investigation comparing specific exercises with sham or usual care treatment exists [85].

On the other hand, the use of analgesics, such as paracetamol, ibuprofen, naproxen, flurbiprofen or ketoprofen, has been suggested to treat pain, promote prostaglandin synthesis and achieve an anti-inflammatory effect [70,81]. Non-steroidal anti-inflammatory drugs (NSAIDs) seem to improve symptoms but do not shorten the course of OSD [71]. In addition, NSAID administration via infiltration has been shown to yield low success rates and, in some cases, may result in atrophy of the subcutaneous adipose tissue [86], the formation of striae in the skin [87] and even tendon ruptures [24,88,89]. All the above is the result of tendon degradation originating from decreased blood supply and altered collagen synthesis. Therefore, prescription of corticosteroids or NSAIDs has been totally discouraged in this type of pathology and should be avoided [90]. In this section, the use of saline and dextrose injections as possible treatment options in ODS should also be highlighted [91]. Saline, despite being recognized as a placebo and pharmacologically inert agent, appears to provide substantial and clinically relevant benefits [92]. In this line, Altman et al. (2016) concluded that its use is effective in the short term (≤3 months) by reducing the pain generated by this type of injury [93]. Despite its possible efficiency, some authors classify it as a treatment option with limited value in real clinical practice [94]. Injected dextrose currently has a lack of high-quality evidence, but despite this it has also been used in the treatment of OSD [95]. A recent study determined its effectiveness as a treatment for this injury by finding significant improvements in the values of the VISA-Patella scores in the intervention group [96]. Although there is scientific evidence that demonstrates the efficiency in the use of both treatment methods, more studies are needed to reinforce their use. Other types of alternative treatments that have been also suggested are the application of autologous conditioned plasma (ACP) [97], manual and electro-acupuncture [83] or dextrose infiltrations [95,98,99], although the latter seem to have a low success rate.

Other forms of treatment could be the use of extracorporeal shock wave therapy [100] or magnetic field therapy [101]. The former has been proven to effectively reduce tendon pain due to its analgesic effects and its remodeling and repair-promoting effects on soft tissue [102]. The latter has been shown to effectively enhance cartilage and bone repair by increasing the bone matrix [101].

Surgery has been suggested only when other types of treatment have previously failed and when bone fragments (intra or extra-tendon) remain after the ossification has been completed [57,102]. Surgery may also be necessary in situations in which non displacement fractures are present [103]. Other techniques and algorithms that have been proposed are percutaneous fixation of the tibial tuberosity [104], extraction of the ossification center, debrination of the tendon or contouring of the process using arthroscopic techniques [105]. Optionally, removal of the tibial tuberosity [106,107] (tibial tuberoplasty) or addition of bone grafts [85,108] have also been considered. Despite the different approaches proposed, each one has advantages and disadvantages [33].

## 8. Practical Applications

The results of the present review suggest that excessive tractional loads should not be applied on the anterior tibial tuberosity through the patellar tendon in children aged 9 to 15. Since complications are very rare (except for pain caused by fragmentation), early detection and differentiation from anterior knee pain are crucial to prevent the complications. For this reason, muscular imbalances that favor higher tension (stiffness) in the patellar tendon should be prevented, particularly in sports with acyclic and explosive components. Moreover, flexibility and muscle rebalancing programs that contribute to reducing tension in the patellar tendon insertion might represent an interesting strategy. For symptom management, a combination of a conservative treatment, training load reduction and limitation of movement patterns that generate tension in the area could contribute to the improvement. All these actions should be aimed at reducing inflammation and symptoms in order to maintain athlete fitness and prevent complications.

Finally, we consider that, due to the scarce existing literature and the contributions given in this work, future research and contributions are necessary to describe the state of the art of this pathology, paying more attention to the type of treatment to be used. Not only from a mere description of the training components but also from the perspective related to the pathology situation. Although it is true that the aspects related to the multifactorial elements are key to understanding this condition, it is necessary to delve much deeper into the treatment itself, both at the preventive and recovery level.

## Data Availability

Not applicable.
